# Fog-Deployable XGBoost Framework for DDoS Detection in IoT Networks

**DOI:** 10.3390/s26196068

**Published:** 2026-09-25

**Authors:** Abeer Albalawi, Linda Mohaisen

**Affiliations:** 1 Department of Information Technology, Faculty of Computing and Information Technology, King Abdulaziz University, Jeddah 21589, Saudi Arabia; lmohaisen@kau.edu.sa; 2 Department of Computer Science, Dar Al-Hekma University, Jeddah 22246, Saudi Arabia

**Keywords:** Internet of Things (IoT), machine learning, DDoS attack, fog computing, XGBoost

## Abstract

The rapid growth of Internet of Things (IoT) deployment has increased exposure to Distributed Denial of Service (DDoS) attacks, while the latency and bandwidth costs of cloud-centric intrusion detection constrain real-time response. This paper presents a fog-based DDoS detection framework that combines XGBoost with information-gain feature selection. The framework uses 23 of the 78 features, a 70.5% reduction, at a measured accuracy cost of 0.007 percentage points. The evaluation used a sample drawn from every file of the CICDDoS2019 release, with benign traffic retained and attack traffic sampled to an attack-to-benign ratio of approximately 10:1. Feature selection, hyperparameter tuning, and threshold calibration were confined to the training partition. Under a random stratified split, the model reached 99.98% accuracy at a 0.058% false-positive rate. Under the capture-day split designated by the dataset authors, accuracy fell to 91.61%, and the false-positive rate rose to 8.82%, equivalent to 8820 false alerts per 100,000 benign flows. Per-request inference on a two-core profile took 1.0 to 5.2 ms over three repeated runs; feature extraction and network transit were not measured. Deployment would require monitoring for distribution shift and periodic retraining.

## 1. Introduction

IoT deployments have grown quickly across business, healthcare, and smart-city settings, where embedding computing and communication in physical devices supports a range of data-driven applications. The same growth widens the attack surface. Because IoT sensors have limited computing power and memory, they are easy targets for DDoS attacks, in which attackers flood a device or network with traffic until normal operation is disrupted [1]. Such attacks disrupt service availability, so detection must be both accurate and timely.

Intrusion Detection Systems (IDSs) have usually been placed in the cloud, where centralized processing can draw on large computing resources. That placement adds latency, because data must cross wide-area networks, and it consumes bandwidth that typical IoT links cannot support [2]. Fog computing addresses both by moving processing closer to the devices that generate the data, at the edge of the network [1,3]. Shifting detection from the cloud to nearby fog nodes reduces delay, lowers bandwidth use, and allows local decisions without a round trip to a remote service.

This paper presents a fog-based DDoS detection framework for resource-constrained IoT networks. Information gain reduces the feature set from 78 to 23, and an XGBoost classifier runs at the fog layer. On a sample drawn from every file of the CICDDoS2019 release, the framework reaches 99.98% accuracy under a random stratified split and 91.61% under the dataset’s designated capture-day split. Under a two-core fog-node budget, per-request inference time ranges from 1.0 to 5.2 ms. Permutation importance, SHAP analysis, and category ablation provide complementary assessments of feature importance and reveal disagreement in how predictive influence is distributed across the selected features. This study makes the following research contributions:

- Establish, on a sample drawn from every file of the CICDDoS2019 release, with all fitting confined to the training partition, that accuracy falls from 99.98% under a random split to 91.61% under the dataset’s designated capture-day split, demonstrating the impact of temporal distribution shift on detection performance.
- Quantify the effect of retaining source port, destination port, and the Inbound flag: their inclusion reduces the error count from 41 to 7 without evidence of improved generalization.
- Reduce the feature set by 70.5% at a measured accuracy cost of 0.007 percentage points and evaluate its serving cost under two resource profiles, with three repeated runs of 3500 requests on the two-core profile, where model inference ranged from 1.0 to 5.2 ms per request at concurrency levels from 1 to 100.
- Quantify predictive feature importance by permutation importance, SHAP values, and category ablation, reporting where the measures disagree rather than relying on a single criterion.

The remainder of the paper is structured as follows. Section 2 reviews related work on fog-based detection, machine learning and deep learning approaches to DDoS attacks, and the difficulty of meeting real-time constraints in IoT settings. Section 3 describes the proposed framework: the system architecture, the feature selection procedure, and the XGBoost configuration. Section 4 reports the evaluation, covering accuracy, computational cost, per-feature impact, generalization across datasets and capture sessions, and comparison with published results. Section 5 concludes and identifies directions for future work.

## 2. Related Work

The Internet of Things (IoT) is now deployed across business, healthcare, and smart-city systems, which has made large volumes of sensor data available for analysis. Most IoT sensors have limited processing power, so they are easily saturated by Distributed Denial of Service (DDoS) traffic, and the resulting outages carry further security exposure. Cloud-based Intrusion Detection Systems (IDS) address this through centralized analysis, but they carry high latency and require sustained backhaul bandwidth that IoT access networks often cannot provide. Fog computing has been proposed as a way to move detection closer to the traffic and reduce both response time and communication load. The location at which computation takes place determines what real-time detection can achieve. Cloud computing is centralized in remote data centers and carries the highest latency and bandwidth costs. Edge computing runs on or near the IoT devices themselves, using hardware such as gateways and routers, where resources are limited. Fog computing occupies the intermediate tier, using micro data centers located within access networks to aggregate traffic and run low-latency analytics without wide-area backhaul. This section reviews ML and DL approaches to DDoS detection across these three tiers. For each study, it reports the dataset, task type (binary or multiclass), and any computational measurements provided by the authors.

### 2.1. Machine Learning for DDoS Detection

Machine learning has become a common approach to DDoS detection and classification in IoT networks, because trained models adapt to traffic patterns that shift over time rather than relying on fixed signatures. The methods applied so far carry recurring limitations: high computational load, class imbalance in the available datasets, and poor scaling as traffic volume grows. Supervised models have performed well on binary classification, where network traffic is labelled either benign or malicious. On CICIoT23, ref. [4] compared XGB, MLP, and RF for IoT-targeted DoS and DDoS detection and found XGB the strongest of the three. Using KNN with selected distance metrics, ref. [5] reported 99.99% accuracy on CICDDoS2019. On IoT-based banking traffic, ref. [6] compared SVM, RF, and KNN, and found SVM to be both the most accurate at 99.5% and the fastest, and concluded it was the better fit for resource-constrained deployment. A broader comparison in [7] evaluated eight algorithms on CICDDoS2019 and placed ensemble methods ahead of individual classifiers, with Random Forest at 98.80% and AdaBoost at 98.76%. That study also reported the trade-off against computational cost, with Decision Tree reaching 98.50% in 2.015 s.

All of these apply supervised learning in a binary setting, so they identify malicious traffic without specifying the attack type, and they depend on training data that does not track changes in traffic behavior. Multi-stage designs address the first of these. The architecture in [8] classifies traffic as benign or malicious, then groups DDoS attacks by transport protocol (TCP, UDP, or mixed), then identifies specific attack types within each group. It reached 99.79% accuracy while cutting processing time by 30% compared with single-stage classifiers.

While traditional machine-learning models perform well in binary DDoS classification, deep-learning models are increasingly used to capture more complex, sequential, and non-linear traffic behavior. LSTM networks capture sequential dependencies in network traffic, and [2] reported 99.97% binary accuracy on that basis. A hybrid in [9] combined the temporal modelling of LSTM with the feature extraction of CNN for real-time classification. [10] tested six architectures (DNMLP, LSTM, Bi-LSTM, GRU, CNN + LSTM and HEM) across four datasets and found LSTM the most consistent, at 99.70%, 99.12%, 94.11% and 99.88% on DDoS-SDN, NSL-KDD, UNSW-NB15 and IoTID20, respectively. In software-defined networking, the Deep Convolutional Neural Network of [11] reached 99.99% on InSDN, 99.97% on CIC-IDS2017 and 99.7% on CICDDoS2019. These models are computationally demanding where low latency matters, and they need large labeled datasets that are expensive to produce.

Beyond individual machine- and deep-learning models, ensemble methods combine multiple classifiers to improve detection performance. A stacking approach over kNN, LR and SGD reached 99.965% in [12]. The two-layer ensemble in [13] passed the outputs of kNN, XGBoost and Gaussian Naive Bayes to a Random Forest meta-classifier and reached a 99.41% detection rate on DS2OS. Running several classifiers at once raises overhead again, which limits the approach in resource-constrained IoT networks.

These limitations recur across all three families. The reported accuracy figures share a further one: they come predominantly from random splits of the same public benchmarks, where training and test traffic are drawn from the same capture period. Section 4 examines whether such accuracy is maintained when the training and test data are separated in time.

### 2.2. Fog Computing as an Enabler for Real-Time Detection

Processing data closer to IoT devices reduces latency and improves scaling for real-time DDoS detection. Entropy-based methods were among the first applied at this layer. In [1], the size of messages arriving at fog nodes is measured against normal traffic patterns, so that deviations surface early. A related approach in [14] combined a randomness measurement of traffic with k-nearest neighbor classification at the fog layer, reporting 100% detection accuracy for TCP and ICMP attacks and 98.7% for UDP. Layered architectures place detection across several tiers. The system in [2] gives fog nodes the local processing work, which cuts the volume sent to the cloud and lowers resource consumption without losing detection accuracy. A four-layer design in [9] separates device, fog, controller, and cloud layers, with the fog layer running Long Short-Term Memory (LSTM) models on live traffic and reaching 98% accuracy. The hybrid framework in [3] pairs signature-based IP blacklisting with XGBoost anomaly detection, reaching 99% accuracy at speeds 6 to 19 times faster than anomaly detection alone. Detected attacks feed back into the signature database, so the blacklist updates itself as new attacks appear. Deep learning has also been placed at this layer. The three-stage hybrid model in [15] uses fog node computation to work around the resource limits of IoT devices, training a Deep Maxout network with the BMUJFO optimization algorithm. It reached 95.26% accuracy, with a reported processing time of 1281.552 s, faster than the traditional optimization approaches the authors compared against. Across these designs, fog computing offers three advantages over cloud-centric detection:

- Lower Latency: Proximity to data sources accelerates anomaly detection.
- Reduced Bandwidth Consumption: Fewer raw packets must traverse the network.
- Localized Decision-Making: Fog nodes can support rapid local responses before an attack reaches the broader IoT infrastructure.

### 2.3. Addressing Real-Time Challenges and Advanced Approaches

Real-time detection in IoT networks faces evolving attack patterns, limited resources and scalability limits, and researchers have approached each of these separately. IoT traffic is dynamic, and its patterns can change substantially over time. These changes, known as concept drift, degrade static detection models. Adaptive systems respond by updating as the distribution shifts. The framework in [16] uses an Accuracy Updated Weighted Probability Averaging Ensemble (AUWPAE) to reweight models as traffic distributions change, reaching 99.33% accuracy on CICIoT2023 and handling both sudden and incremental drift.

Feature selection reduces computational cost without giving up accuracy. The feature extraction methods RSIP and SDFP in [17] improved both classification accuracy and speed, reaching 99.8% on binary classification. A related generalization problem has been studied outside network traffic: in [18], Android APK files are converted to images and classified by deep feature extraction, identifying malware families absent from training at 94.93% precision and 93.75% recall without any dynamic analysis. Beyond adaptation and generalization, scalability and computational efficiency also determine how effectively these systems can extend to large IoT deployments. Fog-layer efficiency has also been addressed outside detection: [19] combines machine learning for link-reliability estimation with multi-objective optimization to select Pareto-optimal paths in an SDN-enabled IoT-fog network, reducing execution time by 13% for a real-time application and packet loss by 41% for a task-offloading application against shortest-path routing. In [10], detection time falls as fog nodes are added: raising the count from one to nine reduced the Communication Behavior Detection Time (CBDT), which is direct evidence for distributed fog architectures. The hybrid fog-cloud architecture in [20] filters traffic at the fog layer and runs distributed machine learning on Apache Spark in the cloud, holding detection accuracy above 98% on NSL-KDD at large deployment scale. A comparable design in [21] combined Spark with TensorFlow on BoT-IoT and reached 98.44% on binary classification.

Several frameworks combine fog computing with learned models. LSTM and CNN models running on fog nodes have reported near-perfect detection rates, with LSTM suited to the sequential structure of attack traffic [9]. Transfer learning fine-tunes a pretrained model on a target dataset, cutting training time and computational cost while holding 99.9% accuracy on multiclass classification [22]. Across benchmark datasets, machine learning and deep learning both report strong results [5,6,7,9,10]. The costs are less often reported. Deep learning models carry compute and memory demands that resist deployment on resource-limited fog nodes, and ensembles add further overhead [12,13]. Many evaluations run in controlled settings rather than operational ones [20], and the computation and latency trade-offs of feature selection for fog deployment remain underexplored [4,15,16].

Table 1 compares machine-learning-based approaches to DDoS detection in IoT networks in terms of deployment layer, detection strategy, dataset, reported accuracy, and real-time suitability. Most approaches report accuracy above 98%, with deployment spanning fog, cloud, and hybrid configurations.

Table 2 provides a corresponding comparison of deep-learning-based approaches. These studies use CNN, LSTM, and hybrid architectures, often deployed at the fog layer to capture complex temporal and spatial patterns in network traffic. Although these approaches generally report high accuracy, their computational demands remain an important consideration for resource-constrained fog environments.

Previous studies have used XGBoost for DDoS detection: as an anomaly detector paired with signature-based methods [3], as a standalone classifier benchmarked against other machine-learning models [4], and as a component of an ensemble framework [13]. However, our study implements XGBoost as the classifier within a fog-based framework, with its primary contribution being a rigorous evaluation protocol that assesses both predictive validity and deployment feasibility. The evaluation draws samples from every file in the CICDDoS2019 release and quantifies duplicate overlap across data splits; excludes identifier and port columns while explicitly measuring the effect of retaining them; confines feature selection, hyperparameter tuning, and threshold calibration to the training partition; evaluates generalization under both random and capture-day splits; and measures the latency, throughput, and resource utilization of the classification stage under concurrent workloads and a constrained CPU budget.

## 3. Proposed Framework

The proposed framework places DDoS classification at the fog layer rather than on the IoT devices themselves. Running the classifier there keeps the computation off devices that have little processing capacity to spare and enables low-latency classification without a round trip to the cloud.

### 3.1. Fog-Based System Architecture

Figure 1 illustrates the proposed framework. In the architectural design, traffic from IoT devices is forwarded to a fog node, where flow features would be extracted and each flow classified as benign or DDoS. Placing the classifier at the fog layer keeps classification off devices that may lack the memory and processing capacity required to run it and avoids a wide-area round trip to a cloud service.

Section 4 evaluates the classification stage using precomputed flow features: the model, its feature set, and the cost of serving it under a fog-node resource budget. Feature extraction from raw packets and the response stage shown in Figure 1 are part of the architectural design but were neither implemented nor measured in this work.

### 3.2. Feature Optimization for Fog Deployment

Information gain was selected as a lightweight and interpretable ranking criterion: it scores each feature independently, so the ranking is inexpensive to compute and can be inspected feature by feature. That same independence means it cannot detect redundancy between features, a limitation recorded in Section 4.8. All 78 features are ranked by information gain and the top 23 are retained, a reduction of 70.5% in the number of features scored per request. Information gain is estimated as the mutual information between each feature and the binary label (benign 0, attack 1) using the k-nearest-neighbor estimator of scikit-learn’s mutual_info_classif (three neighbors, continuous features), computed on a random 200,000-row sample of the training partition with seed 42.

Information gain across the 78 features ranges from 0.2497 for Average Packet Size down to zero. The 23 retained features span scores from 0.2497 to 0.1568, while the highest excluded feature scores 0.1555. Consequently, the cut-off represents a narrow margin rather than a natural break. Section 4.5 reports accuracy against feature count and compares information gain with five alternative criteria and a random baseline, and places the choice of 23 information-gain features in the context of a nested cross-validation over criteria and feature counts confined to the training partition, see Table 3.

This selection preserves detection performance: on the held-out test set of 243,187 flows, the 23-feature set reduced accuracy by only 0.007 percentage points relative to the full 78-feature set.

### 3.3. XGBoost Classification for Fog Computing

The framework uses XGBoost [23] to detect DDoS attacks at the fog layer because of its low per-request inference cost and compact serialized model, both of which are important for deployment on a resource-constrained node. Its accuracy, inference cost, and model size are compared with those of Random Forest and k-NN in Section 4.3. Hyperparameters were selected through randomized search with five-fold cross-validation using the training partition only. The selected hyperparameters are summarized in Table 4, while the search space and sensitivity analysis are reported in Section 4.3.

### 3.4. Fog Computing Implementation Framework

Table 5 states the resource profile the framework targets. The measurements in Section 4.4 were taken on a workstation (Intel Core Ultra 9 275HX, 24 logical cores, 32 GB RAM, Windows 11) with the serving process restricted to two logical cores by CPU affinity and XGBoost limited to two threads, not on dedicated fog hardware. The software stack was Python 3.14.7, scikit-learn 1.9.0, XGBoost 3.4.1, pandas 3.0.5, and NumPy 2.5.2, served by Flask 3.1 under Waitress 3.0.

A container definition targeting the same fog-node profile was built and verified with runtime limits of 2 CPU cores and 2 GB RAM, serving the model with a resident set of 177 MB. The load tests reported in Section 4 were conducted on the host process constrained to two CPU cores through CPU affinity, while memory consumption was monitored against the 2 GB fog-node budget.

## 4. Performance Evaluation

### 4.1. Evaluation Methodology

The evaluation covers two aspects: algorithmic performance on the classification task and computational cost under the fog-node resource budget. Section 4.3 reports the former, and Section 4.4 the latter. Section 4.5, Section 4.6 and Section 4.7 then examine feature impact, generalization across datasets and capture sessions, and comparison with published results.

**Performance Metrics.** Attack flows are treated as the positive class. TP and TN denote correctly classified attack and benign flows, respectively. FP denotes a benign flow incorrectly classified as an attack, whereas FN denotes an attack flow incorrectly classified as benign. The following metrics are reported throughout:

(1) $$\mathrm{Accuracy}=\frac{TP+TN}{TP+TN+FP+FN}$$

(2) $$\mathrm{Precision}=\frac{TP}{TP+FP}$$

(3) $$\mathrm{Recall}=\frac{TP}{TP+FN}$$

(4) $$\mathrm{Specificity}=\frac{TN}{TN+FP}$$

(5) $$\mathrm{FPR}=\frac{FP}{FP+TN}=1-\mathrm{Specificity}$$

(6) $$F_{1}=\frac{2\times \mathrm{Precision}\times \mathrm{Recall}}{\mathrm{Precision}+\mathrm{Recall}}$$

(7) $$\mathrm{MCC}=\frac{TP\times TN-FP\times FN}{\sqrt{\left(TP+FP)(TP+FN)(TN+FP)(TN+FN\right)}}$$

Accuracy alone provides limited information under extreme class imbalance. At the release’s natural attack-to-benign ratio of approximately 618:1, an always-attack classifier would score 99.84%. After stratified sampling to a 10:1 ratio, the evaluation sample is less skewed at 9.27% benign (Section 4.2), but an always-attack classifier would still score 90.7% on it. The false-positive rate and the Matthews correlation coefficient are therefore reported alongside accuracy throughout. Balanced accuracy, ROC-AUC and PR-AUC are reported for the proposed configuration in Section 4.5. All metrics are computed on the held-out test set at the calibrated decision threshold unless a comparison states otherwise.

**Latency and resource metrics.** Model inference time is the time spent inside the XGBoost predict call for one request, measured on the server. Service response time is the wall-clock round trip measured at the client, from sending the request to receiving the label; it includes HTTP handling, queueing, and inference, and excludes network transit because the client and server ran on the same host. Throughput is the number of completed requests divided by the wall-clock duration of a run. Memory is the resident set size of the serving process. For the two-core profile, each reported metric is presented as the mean and standard deviation over three independent runs.

### 4.2. Dataset and Experimental Setup

CICDDoS2019 [24] is used for evaluation. The complete release comprises 70,427,637 flows spanning a wide range of DDoS attack types, making it one of the largest publicly available benchmarks for this task. The release consists of 18 CSV files across two capture days (eleven files recorded on 12 January 2019 and seven on 11 March 2019). All benign flows were retained; attack flows were sampled uniformly at random within each file at a fixed fraction (1.5% to 1.6% of each attack type; WebDDoS 5.0%) with seed 42 and then trimmed with stratification by attack type to a 10:1 ratio, and rows with non-finite Flow Bytes/s or Flow Packets/s were dropped. This procedure yields 1,215,934 records, including 112,731 benign flows (9.27%). Nine columns are excluded: the row index, flow identifier, source and destination addresses, timestamp, and the SimillarHTTP field, together with source port, destination port, and the Inbound flag. This leaves 78 candidate features.

The records are split 80/20 with stratification by attack type, giving 972,747 training and 243,187 test records. All model-development steps are then performed using the training partition. Information-gain ranking is applied to the training data, from which 23 features are selected. Hyperparameter search is conducted using five-fold cross-validation, and the decision threshold is calibrated from five-fold out-of-fold predictions on the training data. Feature ranking is computed once on the training partition rather than refit within each cross-validation fold. The resulting model configuration is then evaluated on the held-out test partition. Where an evaluation uses a different training set, feature selection is refit using the corresponding training data. All experiments use random_state 42 unless stated otherwise.

### 4.3. Algorithmic Performance Analysis

Table 6 compares the three models using the same 23 information-gain features and the same preprocessing. k-NN is evaluated on a smaller sample, for the reason noted below the table. Random Forest and XGBoost are close on accuracy, at 99.97% and 99.98%, while k-NN trails at 99.90%. The separation between them is in deployment cost rather than detection quality. XGBoost’s serialized model is 0.5 MB, compared with 7.8 MB for Random Forest and 28.8 MB for kNN. Furthermore, it scores a batched sample in 0.0009 ms, compared with 0.0049 ms and 0.1147 ms, respectively. Comparable accuracy at a fraction of the memory and compute cost is why XGBoost is the proposed classifier for the fog layer.

k-NN prediction cost grows with the size of the stored training set, so it could not be evaluated at the full scale used for the other models in this experiment. It was therefore restricted to 150,000 training and 50,000 test samples, whereas Random Forest and XGBoost used the complete 972,747/243,187 split. This limitation indicates that k-NN is less suitable for the resource-constrained deployment profile considered in this study.

Table 7 presents the confusion matrix for the proposed configuration. Of the 243,187 held-out test flows, 13 benign flows were incorrectly classified as attacks, while 28 attack flows were incorrectly classified as benign.

Algorithm 1 summarizes the complete detection pipeline. The procedure consists of three phases. Phase 1 reduces the feature set from 78 to 23 by information gain, computed on the training partition only. Phase 2 trains an XGBoost model under five-fold cross-validation and calibrates the decision threshold from out-of-fold predictions. Phase 3 classifies traffic at the fog layer.

The architectural design in Figure 1 places logging, blocking and mitigation after detection. These stages are not implemented or measured in this work; Algorithm 1 describes the detection path that was evaluated.

**Algorithm 1** Fog-based DDoS detection: feature selection and XGBoost Classification**Input:** Training partition $D_{\mathrm{train}}$ with 78 features ($D_{\mathrm{test}}$ held out until final evaluation) 
**Output:** Classification label y∈{Benign,DDoS}, selected feature subset $F^{*}$ (23 features)

**Phase 1: Feature Selection via Information Gain (training partition only)**
1.Initialize empty feature subset $F^{*}\leftarrow \emptyset$2.**for** each feature $f_{i}\in D_{\mathrm{train}}$ (i=1,…,78) **do**3.    Compute $\mathrm{IG}\left(f_{i}\right)=H\left(Y\right)-H\left(Y|f_{i}\right)$, estimated on a random 200,000-row sample of $D_{\mathrm{train}}$4.
**end for**
5.Sort features by $\mathrm{IG}(f_{i})$ in descending order6.**for** each feature $f_{i}$ in sorted order **do**7.    **if** $f_{i}$ is among the top 23 features by $\mathrm{IG}(f_{i})$ rank **then**8.       $F^{*}\leftarrow F^{*}\cup \left\{f_{i}\right\}$9.    **end if**10.
**end for**
11.
// $F^{*}$ contains 23 features (70.5% reduction); no score threshold is applied


**Phase 2: XGBoost Model Training on Fog Node**
12.**for** each sampled hyperparameter configuration *h* **do**13.    **for** k=1,…,5 **do**14.        Split $D_{\mathrm{train}}\left[F^{*}\right]$ into ${\mathrm{Train}}_{k}$ and ${\mathrm{Val}}_{k}$ (stratified)15.        Fit scaler and XGBoost with *h* on ${\mathrm{Train}}_{k}$; evaluate on ${\mathrm{Val}}_{k}$16.    **end for**17.    score(h)← mean accuracy across the five folds18.
**end for**
19.

$h^{*}\leftarrow \arg\max_{h}\mathrm{score}\left(h\right)$ $\left\{n_{\mathrm{estimators}}=200,\;\max\_\mathrm{depth}=6\right\}$

20.M← scaler and XGBoost fitted on all of $D_{\mathrm{train}}\left[F^{*}\right]$ using $h^{*}$21.$p_{\mathrm{oof}}\leftarrow$ out-of-fold probabilities on $D_{\mathrm{train}}\left[F^{*}\right]$ under $h^{*}$, from the same five folds22.

$\tau \leftarrow \arg\max_{\tau \in [0.01,0.99]}\mathrm{Accuracy}\left(y_{\mathrm{train}},\left[p_{\mathrm{oof}}\geq \tau \right]\right)$ {τ=0.395}



**Phase 3: DDoS Classification at the Fog Layer**
23.
DetectDDoS (x)

24.Select $F^{*}$ features from *x* and normalize using statistics fitted on $D_{\mathrm{train}}$25.$\hat{y}\leftarrow 1$ **if** $M.\mathrm{predict}\_\mathrm{proba}(x\left[F^{*}\right])\geq \tau$; **else** $\hat{y}\leftarrow 0$26.**return** $\hat{y}$


**Hyperparameter Sensitivity.** Twenty-five configurations were sampled by randomized search with five-fold stratified cross-validation, scored using accuracy, over n_estimators from 100 to 380 in steps of 20, max_depth from 3 to 11, learning_rate over twenty values from 0.01 to 0.2, and subsample and colsample_bytree over five values each from 0.6 to 1.0. Accuracy varies only between 99.9634% and 99.9836% across the grid and is flat for max_depth of six or more, so the result does not depend on a narrowly tuned configuration.

All configurations in Table 8 are scored at the default 0.5 threshold to isolate the effect of hyperparameter variation. The proposed configuration instead uses the calibrated threshold τ=0.395, obtained from the training partition. Consequently, the configuration with max_depth=6 and n_estimators=200 shows 44 errors here against the 41 reported in Table 6 and Table 7.

**Threshold calibration.**τ is chosen from the grid 0.01 to 0.99 in steps of 0.005 using out-of-fold probabilities from the same five training folds, after the features and hyperparameters are fixed. The lower value is retained in case of ties, and no class weighting is applied during calibration. The threshold is recalibrated in every capture-day and leave-one-attack-type-out run. Calibrating for MCC instead of accuracy selects the same threshold (τ=0.395, 41 errors); calibrating for balanced accuracy gives τ=0.815 and 60 errors; constraining the false-positive rate to at most 0.1% gives τ=0.610 and 47 errors (FPR = 0.040%), while a constraint of 0.05% gives τ=0.940 and 105 errors. The operating point is therefore stable under accuracy and MCC calibration, but shifts when balanced accuracy or explicit FPR constraints are used.

### 4.4. Computational Efficiency for Fog Computing

Table 9 summarizes the measured cost of serving the classification stage under the two-core fog-node budget, as the mean over three independent runs. Inference takes 1.0 to 5.2 ms per request, and mean response time rises with concurrency, from 13.1 ms with a single client to 83.7 ms at fifty and 153.4 ms at one hundred. The p99 rises overall, from 27.7 ms to 217.8 ms (Table 10).

**Load Testing Under Concurrency.**Figure 2 shows the measurement setup. The service ran under Waitress, with the process restricted to two logical cores by CPU affinity and XGBoost limited to two threads; memory was monitored against the 2 GB budget in Table 5 rather than capped. It was tested at seven concurrency levels with 500 requests each on the constrained node (3500 per run, repeated in three independent runs with a fresh warm-up of 100 discarded requests before each) and 1000 requests each on the workstation in a single run (7000 requests). Payloads were drawn from the held-out test split, giving 9.05% benign traffic across twelve attack types; WebDDoS, with 22 rows in the sample, was not drawn. Each request carried one flow as a JSON body of 23 numeric fields (under 1 KB) over HTTP without connection reuse; the server ran Waitress with four worker threads and OMP_NUM_THREADS set to two. All 17,500 measured requests succeeded. The client and service ran on the same host, so the reported times cover request handling, queueing, and inference but exclude network transit.

The measurement procedure was as follows. (1) A pool of 2000 payloads was drawn from the held-out test partition and reused across all concurrency levels and both resource profiles and all runs. (2) An initial 100 requests were issued and discarded so that the reported measurements represented warm-path performance. (3) At each concurrency level, the complete request count was issued through a thread pool of the corresponding size, with each worker measuring the wall-clock round trip at the client. (4) The server returned its own per-stage timings with each response, allowing the residual HTTP, framework, scheduling, and queueing overhead to be estimated as the client-observed round trip minus the recorded server-stage total. (5) Latency percentiles were computed over successful requests only, while failures were counted separately. Throughput was calculated as the number of completed requests divided by the wall-clock duration of the run.

Model inference stays within 1.0 to 5.2 ms at every level. It rises to ten concurrent clients and is then flat, because the server’s thread pool saturates and further requests wait in the queue rather than competing for CPU. At every level, inference is a small fraction of the round trip: at fifty concurrent clients, it accounts for 5.1 ms of 83.7 ms. Run-to-run variation is small at low concurrency (standard deviation of the mean latency below 1.6 ms up to ten clients) and grows once the queue dominates (3.6 ms at fifty and 8.6 ms at one hundred clients). The resident set stays at 302 to 303 MB against the 2 GB budget. Each request carries a single flow, so this figure is not the marginal cost of an additional sample. It includes fixed per-call overhead that is amortized when flows are scored in batches. Table 6 reports a batched inference time of 0.0009 ms per sample for the same model, measured using a batch size of 20,000 samples.

Percentiles are computed over successful requests within each run and averaged across runs. p50 is the median, and p95 and p99 are the latency values below which 95% and 99% of requests complete. They are reported alongside the mean because the two can differ substantially: at five concurrent clients, the mean is 12.5 ms against a median of 7.8 ms, while at 100 clients, the mean is 153.4 ms and the median is 169.1 ms.

### 4.5. Feature Impact Analysis for Fog Optimization

Feature impact is assessed through permutation importance [25] and SHAP values [26], computed on the held-out test set. Of the 23 selected features, one belongs to the Connection category, seventeen to Packet Flow, and five to Timing.

**Feature-Set Comparison.** Reducing the feature set from 78 to 23 costs 0.007 percentage points of accuracy (41 errors against 25 out of 243,187 test samples), so the reduction preserves detection performance. See Table 11. Retaining the port columns and the Inbound flag lowers the error count further, but they are excluded from the reported configuration, since port fields identify where traffic was sent rather than how it behaved. Across five independent runs, scored at the default 0.5 threshold, accuracy is 99.9816%±0.0003 percentage points (95% CI 99.9813% to 99.9819%), with specificity 99.9148% and MCC 0.9989. Each run redraws the train/test split and the XGBoost seed, with the hyperparameters of Table 4 held fixed.

For the reported configuration at the calibrated threshold, specificity is 99.9423%, the Matthews correlation coefficient is 0.998998, and balanced accuracy is 99.9648%, with ROC-AUC and PR-AUC both above 0.99999.

Table 8, Table 12 and Table 13 and the feature-count analysis below score every configuration at the default 0.5 threshold, so that only the factor under study varies. The calibrated τ is derived from one specific configuration and does not transfer to the others. This is why the proposed configuration appears as 44 errors in those comparisons against the 41 reported here, and the full feature set as 21 against 25.

**Predictive Feature Importance.** Importance was quantified directly by permutation importance and SHAP values on the held-out test set, and by removing each category and re-measuring accuracy. Permutation importance and category ablation agree that the single Connection feature is the most critical. It has the highest permutation importance at 0.0793 against 0.0099 for the next feature, and removing its category raises the error count from 44 to 1048 and the false-positive rate from 0.044% to 3.468%. SHAP ranks differently, placing Min Packet Length highest at 5.20 against 1.31 for the Connection feature (Figure 3). The two measures capture different properties: SHAP reflects how strongly a feature moves individual predictions, while permutation reflects how much the model degrades without it. Min Packet Length has the larger SHAP magnitude but a permutation importance of only 0.0062, which suggests that correlated or redundant features can compensate for its loss, whereas the Connection feature appears to have substantially less redundancy within the selected set. Removing all five Timing features leaves performance essentially unchanged, at 43 errors against a baseline of 44. Three of the twenty-three selected features are exact functions of others in the set: Avg Fwd Segment Size is identical to Fwd Packet Length Mean, Subflow Fwd Bytes is identical to Total Length of Fwd Packets, and Packet Length Variance is the square of Packet Length Std. All three score zero importance under both measures, a limitation of univariate selection.

**Comparison with Alternative Selection Criteria.** Six criteria spanning filter, embedded, and wrapper families were first compared on the test partition with the classifier and its hyperparameters held fixed, so that only the feature subset varies (Table 13a). All principled criteria fall within 99.982% and 99.991% accuracy, and all decisively outperform random selection. Information gain is not the strongest: the ANOVA F-test attains 22 errors against 44, a difference that a paired exact McNemar test on the 243,187 test rows confirms (26 rows where information gain errs alone against 4 the reverse, $p=5.9\times 10^{-5}$; bootstrap 95% interval on the difference 12 to 33 errors, 10,000 paired resamples, percentile bounds, no multiple-comparison correction), but which amounts to 0.009 percentage points.

To select the criterion and the feature count without reference to the test partition, both were then chosen by nested cross-validation confined to the training partition, following [27]: five outer folds over the 972,747 training rows, and within each outer-training part five inner folds in which the six criteria ranked the features and were scored by MCC at k∈{10,15,20,23,25,30,40,50,78}; the configuration selected in each outer fold was scored once on that fold’s held-out rows, alongside the deployed 23-feature information-gain configuration (Table 13b). The procedure did not converge on a single criterion: it selected recursive elimination, XGBoost gain, L1, and ANOVA in different folds, at 40 or 50 features, and the selected feature sets overlapped only moderately across folds (mean Jaccard 0.60), whereas the 23 information-gain features were identical in every fold and across sampling seeds 42 to 46 (Jaccard 1.00).

Over the 972,747 held-out rows, the fold-selected configurations made 163 errors against 205 for information gain (paired exact McNemar $p=4.7\times 10^{-5}$), a difference of 0.004 percentage points. These results show that information gain provides performance close to the nested-selected configurations while retaining a compact and stable 23-feature set.

The number of retained features was likewise examined at ten values of *k*: 6240 errors at five features, 1109 at fifteen, 41 at twenty, 44 at twenty-three, 24 at fifty and 21 at seventy-eight. Accuracy rises steeply to twenty features and is essentially flat thereafter, placing the 23-feature configuration within the performance plateau.

### 4.6. Generalization and Temporal Evaluation

**Same-Methodology Reproductions and Capture-Day Evaluation.** Generalization was assessed in two ways. First, the same methodology was applied to three further datasets to examine whether the pipeline could be reproduced across datasets with different feature schemas. Because the feature schemas differ, these experiments represent same-methodology reproductions rather than transfers of the trained model. The IoT-23 evaluation uses Zeek conn.log features on a multi-scenario sample rather than the full release and classifies all 34,966 test samples correctly. Given the different feature extraction and the restricted sample, this result describes the evaluated sample rather than general performance on IoT-23.

Second, and more demanding, the model was trained on the capture designated for training by the dataset authors and tested on the separate capture recorded eight weeks later. The same 1,215,934-row sample was partitioned by capture day rather than at random: the 12 January capture (843,549 rows; 56,425 benign and 787,124 attack) was used for training, and the 11 March capture (372,385 rows; 56,306 benign and 316,079 attack) for testing. Feature ranking, hyperparameter search, and threshold calibration were refit using the January rows only. Accuracy falls from 99.98% to 91.61%, while the false-positive rate rises from 0.058% to 8.82%, revealing a degradation that is not apparent under the random split. This experiment therefore represents a within-release temporal shift test rather than an independent operational deployment evaluation.

**False-Alert Rate.** At the capture-day false-positive rate, the detector would generate 8820 false alerts per 100,000 benign flows, compared with 58 under the random split. Precision depends on attack prevalence; with recall *R* and false-positive rate FPR held at their measured values and prevalence π, precision = πR/[πR+(1−π)FPR]. At prevalences of 0.1%, 1%, 5%, and 10%, the corresponding capture-day precision values are 1.0%, 9.5%, 35.4%, and 53.6%, compared with 63.4%, 94.6%, 98.9%, and 99.5% under the random split. Under benign-dominated traffic, these results show that the false-positive rate is a more informative indicator of operational usability than overall accuracy, see Table 14.

Rows other than the first two use different feature schemas and are reproductions of the methodology on independent data, not evaluations of the trained CICDDoS2019 model.

**Duplicate-Aware Sensitivity Analysis.** Because exact duplicate records can make random train/test partitions optimistic, we additionally performed a duplicate-aware sensitivity analysis to quantify the extent to which the reported results depend on cross-partition duplication. The original split is retained for direct comparison with the main evaluation, while the duplicate-aware analysis provides a more conservative assessment on unique flows. In the original random split, exact duplicate rows, identical across all 78 retained features, account for 829,464 of the 1,215,934 sampled rows (68.2%). In addition, 172,931 of the 243,187 test records (71.1%) have an exact copy in the training partition.

After duplicate removal, 386,470 unique rows remained, of which 309,176 were used for training and 77,294 for testing. When the reported model was evaluated only on the 70,256 test records with no exact duplicates in the training partition, it made 26 errors (99.963% accuracy; 0.027% false-positive rate). Retraining the same pipeline on the unique rows resulted in 147 errors on the 77,294-row test set (99.81% accuracy; 0.28% false-positive rate; MCC 0.995), with 19 of the 23 selected features unchanged. These results indicate that the random-split accuracy of 99.98% is optimistic for previously unseen flows by approximately 0.2 percentage points.

On the capture-day split, 49.2% of the March rows have an exact copy in the January capture, with substantial variation across attack types (NetBIOS 96.8%, Portmap 97.3%, LDAP 92.5%, MSSQL 71.5%, and SYN 0.1%). On the remaining rows, the capture-day model achieves 80.7% accuracy, with an overall recall of 0.753, SYN recall of 0.552, and Portmap recall of 0.870. Retraining on unique rows yields 88.8% to 92.1% accuracy across two hyperparameter choices, with SYN recall ranging from 0.23 to 0.47 and a false-positive rate of 3.9% to 4.1%.

The duplicate-aware analysis also changes the interpretation of attack-specific results. The apparent detection of Portmap, the only attack type absent from the training capture, is largely associated with Portmap flows that are identical in feature space to flows of other attack types in the training capture; it therefore does not provide evidence of generalization to a novel attack type. In contrast, the degradation in SYN detection and the elevated benign false-positive rate persist after duplicate removal, indicating that these effects are associated with the temporal shift rather than exact cross-capture duplication.

**Robustness to Unseen Attack Types.** Four attack types (WebDDoS, Portmap, MSSQL, and TFTP), spanning the smallest and largest classes, were each removed entirely from training, with feature selection refit on the reduced training set so that the excluded type could not influence it. Where the held-out set is large enough to measure reliably, the withheld types remain detectable at close to full recall: Portmap, MSSQL, and TFTP all exceed 0.99. WebDDoS is the exception, with a recall of 0.25. The release contains only 439 WebDDoS flows in total, four of which fall in the test split, and the model reaches only 0.50 recall on those same four samples when WebDDoS is present in training. The WebDDoS result is therefore indicative rather than statistically reliable.

The capture-day evaluation provides a complementary assessment of temporal robustness. Across the seven attack types present in the testing capture, recall is 1.0000 for UDP (n=61,333) and UDPLag (n=24), 0.9999 for LDAP (n=30,199), MSSQL (n=90,679), and NetBIOS (n=57,136), and 0.9965 for Portmap (n=2889). SYN alone falls to 0.6445 over 73,819 samples. Portmap is the single type absent from the training capture; however, its near-perfect recall is largely explained by duplicate flows, as shown in the duplicate-aware sensitivity analysis above, and therefore does not provide evidence of generalization to a novel attack type. In contrast, SYN is well represented in training but performs substantially worse in the later capture, and this degradation persists after duplicate removal. This result indicates that temporal distribution shift within a known attack type remains an important limitation. Sustained deployment would therefore require monitoring for distribution shift and periodic retraining.

Zero-shot methods such as [18], which detect malware families absent from training, address the complementary case of genuinely novel threats. The leave-one-attack-type-out results reported here cover four withheld types and should not be interpreted as evidence that arbitrary novel or zero-day attacks would be detected, see Table 15.

### 4.7. Comprehensive Literature Comparison

Table 16 compares the proposed framework with published results on CICDDoS2019. Under a random stratified split, it reaches 99.98% accuracy, against 99.68% for the RBFN, SVM and K-means hybrid of [28], approximately 99% for the signature and XGBoost hybrid of [3], 98.80% for the PCA-reduced Random Forest of [7], 98.00% for the hybrid LSTM-CNN model of [9], and 95.26% for the optimized Deep Maxout model of [15]. Under the dataset’s designated capture-day split, it reaches 91.61%. That second figure has no counterpart in the table because none of the cited studies report a temporally separated split, which is one reason both protocols are given here separately. The nearest of these figures, 99.68% in [28], comes from a four-class problem restricted to SYN, UDP, and UDP-Lag traffic over approximately one million SMOTE-balanced samples, against the binary task and thirteen attack types evaluated here. More generally, the comparison is indicative rather than controlled. Most of the cited studies do not state their evaluation protocol, sample size, or class balance, so Table 16 records those conditions where they are available, and the note below distinguishes measured rows from published ones. None of the cited studies report a false-positive rate, which is the figure that governs whether a detector is deployable on benign-dominated traffic.

Only the first two rows were measured in this study. The remainder are figures as published by their authors, under protocols that in most cases are not stated, so the comparison is indicative rather than controlled.

### 4.8. Limitations

Duplicate Flows. Exact duplicates were removed only in the sensitivity analysis of Section 4.6; the main results use the sample as drawn, where 71.1% of test records have an identical training copy. Near-duplicates and same-session flows were not assessed.

Temporal Stability. Accuracy falls from 99.98% to 91.61% between capture sessions. Some attack-specific results are affected by cross-capture duplication, while SYN degradation persists after duplicate removal, indicating temporal distribution shift.

Deployment Measurement. The fog node was emulated by restricting the serving process to two CPU cores on a 24-core workstation. Only a single node was evaluated, and the reported measurements were obtained from the host process rather than dedicated fog hardware.

Latency Scope. Reported latency covers classification only and excludes network transit, packet capture, flow aggregation, and feature extraction. Several retained features, including Flow IAT Max and Flow IAT Std, are defined over a completed flow. Accordingly, for the evaluated feature representation, the earliest point at which classification can be performed is after the required flow features have become available. The reported values therefore do not represent end-to-end detection latency.

Detection Scope. The framework performs attack detection but does not implement mitigation. Application-layer attacks that resemble legitimate traffic at the flow level fall outside its detection scope.

## 5. Conclusions

This paper presents a fog-based DDoS detection framework that combines information-gain feature selection with an XGBoost classifier at the fog layer. On a 1,215,934-record sample drawn from every file of the CICDDoS2019 release, reducing the feature set from 78 to 23 costs 0.007 percentage points of accuracy, and the resulting model reaches 99.98% accuracy with a 0.058% false-positive rate under a random stratified split. Across three repeated runs on the two-core profile and one run on the workstation, every request succeeded; on the two-core profile, model inference took 1.0 to 5.2 ms per request with a resident set of 302 to 303 MB. Three additional findings provide further context for these results. Under the capture-day split designated by the dataset authors, accuracy falls to 91.61%, and the false-positive rate rises to 8.82%. The degradation demonstrates the impact of temporal distribution shift on detection performance, indicating that sustained deployment would require drift monitoring and periodic retraining. The 23-feature information-gain set maintained high predictive performance and remained stable across the evaluated resamples. Duplicate-aware analysis reduced the random-split accuracy to 99.81%, indicating that duplicate records can make random-split performance appear more optimistic. The fog node was emulated on a workstation rather than deployed on physical fog hardware, and the reported latency covers the classification stage only, without feature extraction or network transit. Evaluation on physical fog hardware is therefore required to validate these deployment measurements. Future work should also investigate correlation-aware feature selection and adaptive retraining strategies to respond to drift between capture sessions. The results show that near-perfect random-split accuracy alone is insufficient to establish deployment readiness; duplicate-aware evaluation, temporal generalization, and resource-aware evaluation are also important when assessing DDoS detection at the fog layer.

## Figures and Tables

**Figure 1:**
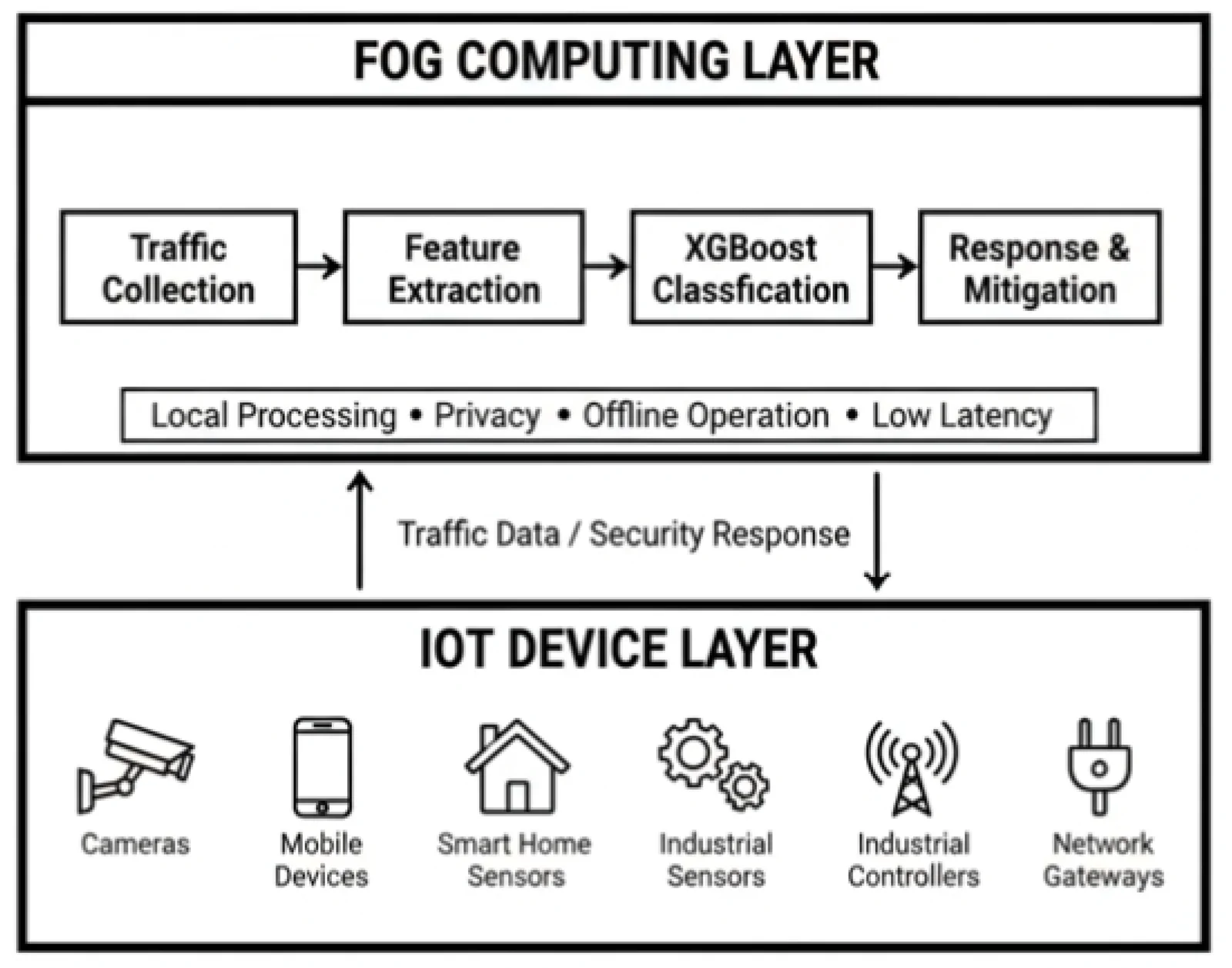
The proposed framework.

**Figure 2:**
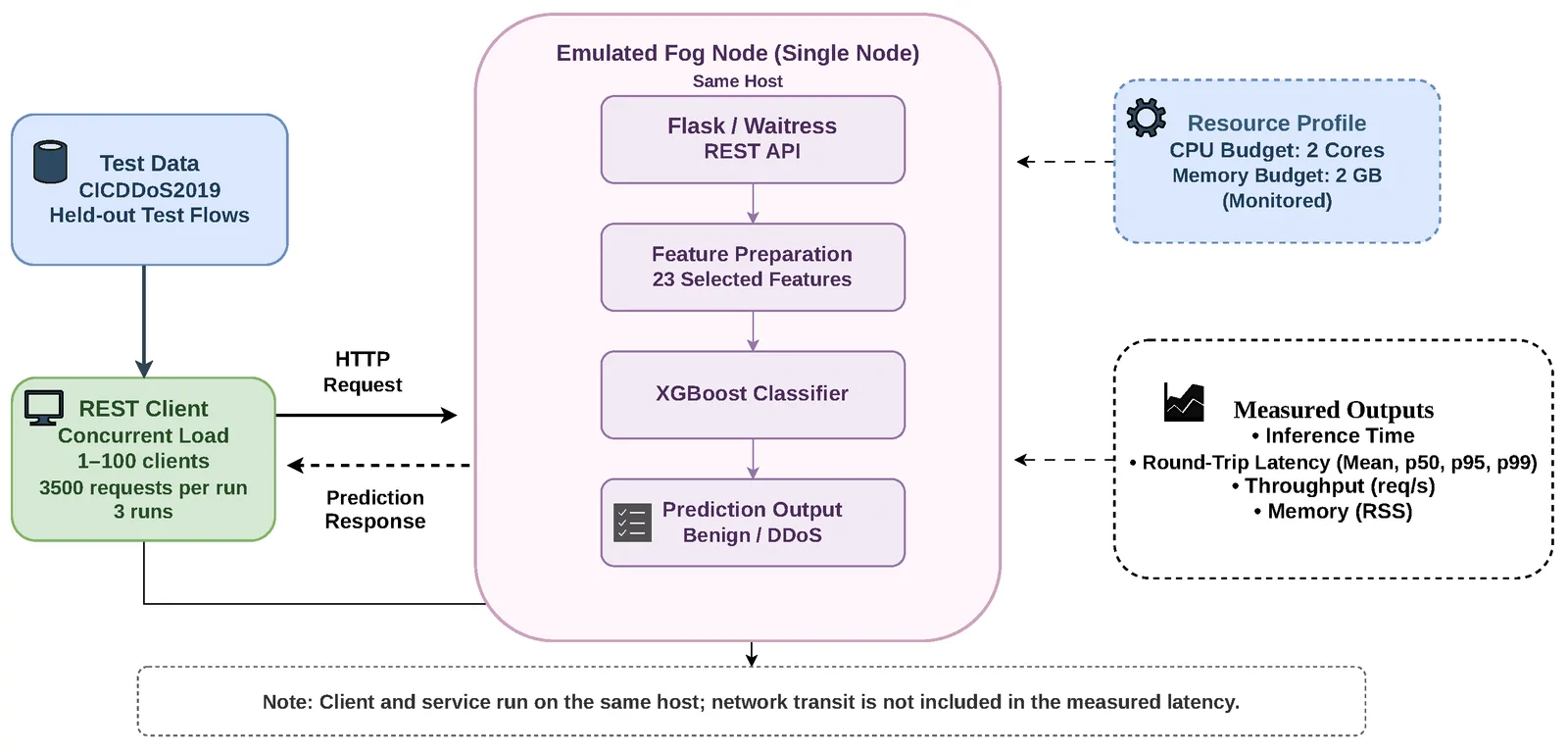
Load-test measurement setup on the emulated two-core fog node.

**Figure 3:**
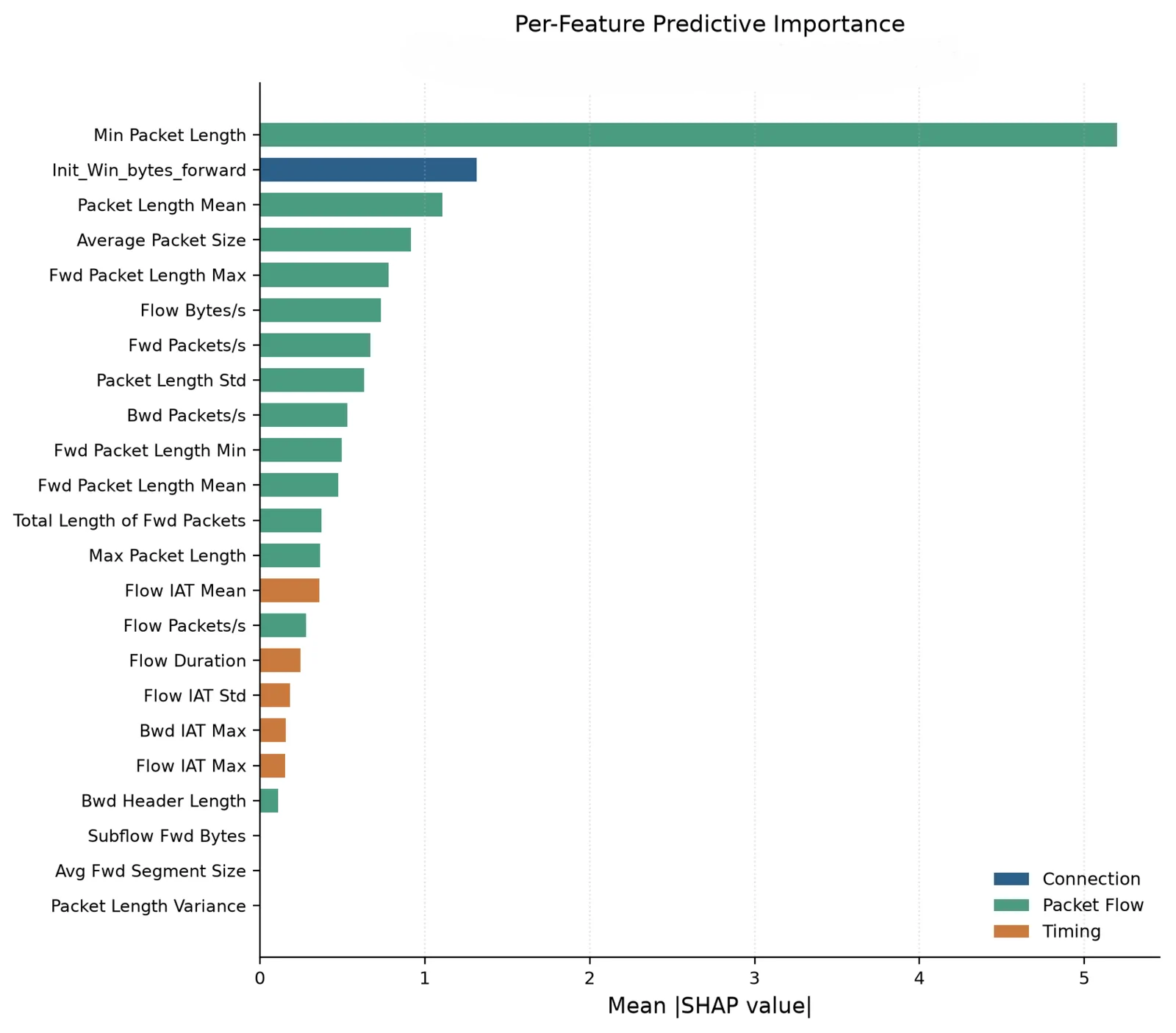
Per-feature predictive importance based on mean absolute SHAP values.

**Table 1:** Comparative analysis of machine-learning models for DDoS detection.

Ref	Year	Attack	Layer	Strategy	Dataset	Accuracy	Real-Time Suitability
[1]	2021	DDoS	Fog Layer	Entropy-based detection using KNN	Meteorology data	99%	Yes
[5]	2021	DDoS	Fog Layer	Statistical analysis- based detection	CICDDoS2019	99.99%	No
[12]	2024	DDoS	Fog Layer	Hybrid ensemble learning approach	CICIoT2023	99.9%	Yes
[21]	2021	DDoS	Fog Layer	CNN–LSTM hybrid model	BoT-IoT	98.44%	No
[6]	2022	DDoS	Not specified	Supervised ML approach	Banking Dataset	SVM: 99.5% RF: 98.74% KNN: 97.8%	Yes
[17]	2022	DDoS	SDN-enabled IoT	Adaptive ensemble voting (SVM + RF)	Environment-Specific Dataset	99.8%	No
[13]	2021	Intrusion Detection	Fog Layer	Ensemble learning (KNN, XGBoost, NB)	UNSW-NB15, DS2OS	UNSW-NB15: 93.21% DS2OS: 99.41%	Yes
[20]	2025	Botnet-based DDoS	Hybrid	Hybrid ML with Apache Spark	NSL-KDD	99.67% (Fog) 98.34% (Cloud)	No
[7]	2025	DDoS	Cloud	Supervised ML approach	CICDDoS2019	98.80%	Yes
[8]	2022	DDoS	Cloud	Ensemble (XGB, LGBM, CatBoost, NB)	CICDDoS2019	99.7% → 99.94%	No
[3]	2020	Botnet-based DDoS	Fog	XGBoost + Signature IDS	BoT-IoT	99%	Yes
[4]	2024	DDoS	Not specified	XGBoost, RF with PSO	IoT-23	98.77%	No

**Table 2:** Comparative analysis of deep-learning models for DDoS detection.

Ref	Year	Attack	Layer	Strategy	Dataset	Accuracy	Real-Time Suitability
[2]	2020	DDoS	Fog (SDN)	Deep CNN (DCNN)	CICDDoS2019	99.97%	Yes
[16]	2024	DDoS	Fog	Drift-Adaptive AUWPAE	CICIoT2023	99.33%	No
[11]	2023	DDoS	Edge (SDN)	DCNN	InSDN, CIC-IDS2017, CICDDoS2019	99.99% 99.97% 99.77%	Yes
[9]	2021	DDoS	Fog	LSTM (Anomaly) + CNN (Classification)	CICDDoS2019	LSTM: 98% CNN: 86%	Yes
[22]	2021	Anomalies	Cloud	CNN (1D, 2D, 3D), Transfer Learning	BoT-IoT, IoT-23, IDS2020	99.9%	Yes
[10]	2022	DDoS	Fog	LSTM, Bi-LSTM, GRU, CNN-LSTM, DNMLP	NSL-KDD, UNSW-NB15, IoTID20, DDoS-SDN	99.70%	No
[15]	2024	DDoS	Fog	Deep Maxout + DBN + BMUJFO Optimization	CICDDoS2019, UNSW-NB15	95.26%	No

**Table 3:** Selected features for fog processing.

Category	Features	Description
Timing	Flow IAT Mean, Flow IAT Std, Flow IAT Max, Flow Duration, Bwd IAT Max	Inter-arrival time patterns
Packet Flow	Flow Bytes/s, Min Packet Length, Fwd Packets/s, Fwd Packet Length Min, Packet Length Std, Average Packet Size, Bwd Packets/s, Total Length of Fwd Packets, Packet Length Mean, Fwd Packet Length Mean, Bwd Header Length, Max Packet Length, Fwd Packet Length Max, Flow Packets/s, Avg Fwd Segment Size, Subflow Fwd Bytes, Packet Length Variance	Packet size, volume, and rate characteristics
Connection	Init_Win_bytes_forward	Connection establishment characteristic

**Table 4:** XGBoost hyperparameters.

Parameter	Value	Notes
n_estimators	200	
max_depth	6	Performance is flat for max_depth ≥6
learning_rate	0.18	
subsample	0.6	60% of training rows sampled per tree
colsample_bytree	1.0	All 23 features available to each tree

**Table 5:** Fog-node resource profile.

Component	Specification	Implementation Detail
Processing	2 CPU cores	Enforced by CPU affinity; XGBoost limited to two threads
Memory	2 GB RAM	Budget monitored, not enforced
Deployment	Containerized services	Flexible fog node deployment
Interface	RESTful API	Flask application served by Waitress

**Table 6:** Algorithm performance comparison.

Algorithm	Accuracy	F1	FPR	Inference (ms/Sample, Batched)	Model Size (MB)
KNN	99.90%	99.94%	0.4454%	0.1147	28.8
Random Forest	99.97%	99.98%	0.0710%	0.0049	7.8
XGBoost (Proposed)	99.98%	99.99%	0.0577%	0.0009	0.5

**Table 7:** Confusion matrix results.

Actual/Predicted	Benign	DDoS
Benign	22,533	13
DDoS	28	220,613

**Table 8:** Hyperparameter sensitivity analysis.

max_Depth	n_Estimators	Accuracy	Errors
3	100	99.9634%	89
3	200	99.9753%	60
3	340	99.9807%	47
6	100	99.9823%	43
6	200	99.9819%	44
6	340	99.9836%	40
9	100	99.9811%	46
9	200	99.9815%	45
9	340	99.9815%	45
11	100	99.9819%	44
11	200	99.9823%	43
11	340	99.9819%	44

**Table 9:** Fog computing performance metrics.

Metric	Value	Notes
Model computation time	1.0–5.2 ms per request	Single flow per request; batched cost in Table 6
Service response time	13.1 ms at one client, 153.4 ms at one hundred	Increases with concurrency; percentiles in Table 10
Memory usage	302–303 MB	Below the 2 GB evaluation budget
Throughput	76–556 req/s	One to one hundred concurrent clients

**Table 10:** API performance under concurrency (two-core fog node; mean ± SD over three runs).

Concurrent Clients	Throughput (req/s)	Mean (ms)	p50	p95	p99	Inference (ms/Request)	RSS (MB)
1	76.0±0.9	13.1±0.2	15.1	22.2±5.0	27.7±0.2	1.01±0.02	302
2	147.2±7.4	13.5±0.7	14.0	31.5±2.9	46.6±9.4	1.81±0.20	302
5	391.0±28.9	12.5±0.9	7.8	30.8±1.2	39.0±5.8	3.24±0.18	302
10	496.8±51.8	19.5±1.6	16.2	37.6±5.1	71.7±25.8	5.19±0.41	302
25	549.4±42.8	43.4±3.5	41.9	63.9±6.4	80.8±18.7	4.95±0.48	303
50	555.8±25.6	83.7±3.6	84.3	107.9±10.9	114.0±10.3	5.13±0.26	303
100	545.1±38.5	153.4±8.6	169.1	208.2±21.8	217.8±20.5	5.14±0.28	303

**Table 11:** Feature-set comparison on the CICDDoS2019 sample.

Configuration	Features	Accuracy	Precision	Recall	F1	FPR	Errors
Full feature set	78	99.990%	99.993%	99.996%	99.994%	0.067%	25
Information gain (proposed)	23	99.983%	99.994%	99.987%	99.991%	0.058%	41
With identifier columns	81	99.997%	99.999%	99.998%	99.998%	0.009%	7

**Table 12:** Predictive importance by feature category.

Category	Features	∑ Permutation Importance	∑ Mean |SHAP|	Mean |SHAP| per Feature	Errors When Category Removed
Connection	1	0.0793	1.31	1.31	1048
Packet Flow	17	0.0189	12.66	0.74	201
Timing	5	0.0004	1.10	0.22	43

**Table 13:** Feature-selection criterion and feature-count analysis.

**(a) Comparison of Feature-Selection Criteria (23 Features Each)**						
**Selection Criterion**		**Accuracy**	**F1**	**FPR**	**Errors**	**Overlap with IG**
ANOVA F-test		99.9910%	99.9950%	0.0399%	22	10/23
L1 logistic regression		99.9889%	99.9939%	0.0488%	27	6/23
Chi-squared		99.9873%	99.9930%	0.0444%	31	9/23
XGBoost gain		99.9864%	99.9925%	0.0444%	33	9/23
Recursive feature elimination		99.9864%	99.9925%	0.0532%	33	12/23
Information gain (proposed)		99.9819%	99.9900%	0.0444%	44	23/23
Random selection		99.6920%	99.8305%	2.8032%	749	8/23
**(b) Nested Selection of Feature-Selection Criterion and Feature Count Within the Training Partition**						
**Outer fold**	**Selected (max MCC)**	**Errors**	**Selected (1-SE Rule)**	**Errors**	**Information Gain, 23**	**Held-Out Rows**
0	RFE, k=50	36	ANOVA, k=40	36	50	194,550
1	XGBoost gain, k=50	29	XGBoost gain, k=30	27	34	194,550
2	L1, k=40	33	ANOVA, k=25	31	37	194,549
3	ANOVA, k=40	36	L1, k=15	50	47	194,549
4	ANOVA, k=50	29	ANOVA, k=23	30	37	194,549
Total		163		174	205	972,747

Note: Five outer folds over the training partition; inner five-fold selection by MCC with the classifier and hyperparameters of Table 4 held fixed and the 0.5 threshold; the test partition was not used. Feature-set stability across outer folds (mean pairwise Jaccard): information gain 1.00, selected configurations 0.60 (max MCC) and 0.44 (1-SE).

**Table 14:** Same-methodology reproductions and capture-day evaluation results.

Dataset	Evaluation Protocol	Test Samples	Accuracy	Precision	F1	FPR
CICDDoS2019	Random stratified split	243,187	99.9831%	99.9941%	99.9907%	0.0577%
CICDDoS2019	Designated capture-day split	372,385	91.6124%	98.3153%	94.8868%	8.8197%
CICDDoS2019 + CICIDS2017	Random stratified split	200,001	99.6140%	99.6051%	99.7400%	1.1341%
CICIoT2023	Dataset-defined split	56,331	99.9964%	100%	99.9981%	0.0%
IoT-23	Random stratified split	34,966	100%	100%	100%	0.0%

**Table 15:** Leave-one-attack-type-out validation.

Held-Out Attack Type	Test Samples	Recall When Unseen	Recall When Seen
WebDDoS	4	0.2500	0.5000
Portmap	578	1.0000	1.0000
MSSQL	32,318	0.9999	0.9999
TFTP	63,173	0.9999	1.0000
Portmap (capture-day)	2889	0.9965	Absent from training capture

**Table 16:** Comprehensive performance comparison with the literature.

Study	Method	Dataset	Samples	Task	Protocol	Accuracy	F1	FPR
This work	XGBoost + information gain (23 of 78)	CICDDoS2019	1,215,934	Binary	Random stratified split	99.98%	99.99%	0.058%
This work	XGBoost + information gain (23 of 78)	CICDDoS2019	1,215,934	Binary	Designated capture-day split	91.61%	94.89%	8.82%
[28]	RBFN + SVM, K-means	CICDDoS2019	∼1,000,000(SMOTE-balanced)	4-class	Random split	99.68%	99.55%	–
[7]	Random Forest (PCA, 24 features)	CICDDoS2019 (custom subset)	∼1,000,000	4-class	60/40 split	98.80%	–	–
[9]	Hybrid LSTM-CNN	CICDDoS2019	–	Binary	–	98.00%	–	–
[15]	Optimized Deep Maxout (BMUJFO)	CICDDoS2019	–	Binary	–	95.26%	–	–
[3]	Signature + XGBoost hybrid	CICDDoS2019	–	Binary	–	∼99%	–	–

Note: "–" indicates that the metric or experimental detail was not reported in the original study.

## Data Availability

The dataset used in this study (CICDDoS2019) is publicly available from the Canadian Institute for Cybersecurity (CIC). The row indices of the evaluation sample and the results files are available from the corresponding author on request.
